# External validation of five predictive models for postoperative cardiopulmonary morbidity in a Chinese population receiving lung resection

**DOI:** 10.7717/peerj.12936

**Published:** 2022-02-09

**Authors:** Guanghua Huang, Lei Liu, Luyi Wang, Zhile Wang, Zhaojian Wang, Shanqing Li

**Affiliations:** Department of Thoracic Surgery, Peking Union Medical College Hospital, Chinese Academy of Medical Sciences and Peking Union Medical College, Beijing, China

**Keywords:** Validation, Predictive models, Morbidity, Lung cancer

## Abstract

**Background:**

No postoperative cardiopulmonary morbidity models have been developed or validated in Chinese patients with lung resection. This study aims to externally validate five predictive models, including Eurolung models, the Brunelli model and the Age-adjusted Charlson Comorbidity Index, in a Chinese population.

**Methods:**

Patients with lung cancer who underwent anatomic lung resection between 2018/09/01 and 2019/08/31 in our center were involved. Model discrimination was assessed by the area under the receiver operating characteristic curve. Model calibration was evaluated by the Hosmer–Lemeshow test. Calibration curves were plotted. Specificity, sensitivity, negative predictive value, positive predictive value and accuracy were calculated. Model updating was achieved by re-estimating the intercept and/or the slope of the linear predictor and re-estimating all coefficients.

**Results:**

Among 1085 patients, 91 patients had postoperative cardiopulmonary complications defined by the European Society of Thoracic Surgeons. For original models, only parsimonious Eurolung1 had acceptable discrimination (area under the receiver operating characteristic curve = 0.688, 95% confidence interval 0.630–0.745) and calibration (*p* = 0.23 > 0.05) abilities simultaneously. Its sensitivity, specificity, positive predictive value, negative predictive value and accuracy were 0.700, 0.649, 0.153, 0.960 and 0.653, respectively. In the secondary analysis, increased pleural effusion (*n* = 94), which was nonchylous and nonpurulent, was labeled as a kind of postoperative complication. The area under the receiver operating characteristic curve of the models increased slightly, but all models were miscalibrated. The original Eurolung1 model had the highest discrimination ability but poor calibration, and thus it was updated by three methods. After model updating, new models showed good calibration and small improvements in discrimination. The discrimination ability was still merely acceptable.

**Conclusions:**

Overall, none of the models performed well on postoperative cardiopulmonary morbidity prediction in this Chinese population. The original parsimonious Eurolung1 and the updated Eurolung1 were the best-performing models on morbidity prediction, but their discrimination ability only achieved an acceptable level. A multicenter study with more relevant variables and sophisticated statistical methods is warranted to develop new models among Chinese patients in the future.

## Introduction

Lung cancer has been the leading cause of cancer death in China (Cao & Chen, 2019). Lung cancer incidence may continuously increase in the next ten years (Cao & Chen, 2019). As modern thoracic surgery approaches have been widely adopted and improved by medical practitioners, video-assisted thoracoscopy for anatomic pulmonary resection has been preferred for patients with early-stage non-small cell lung cancer (Hirsch et al., 2017). Preoperative comorbidities, such as coronary artery disease and cerebrovascular disease, are critical determinants for prognosis, as they could significantly increase the perioperative risk (Iachina et al., 2015; Sandri et al., 2017). In this context, there has been a growing demand for a risk-adjusted model to assess the preoperative risk stratification of patients undergoing thoracic surgery.

Several models have been developed in the past few decades, including the Brunelli model, Thoracoscore and the European Society Objective Score (Taylor et al., 2020). Recently, Eurolung models were developed and updated to predict postoperative cardiopulmonary morbidity and perioperative mortality among patients receiving anatomic lung resections based on the European Society of Thoracic Surgeons database (Brunelli et al., 2017). The model came up with a series of numerical or categorical variables, including demographic characteristics, comorbidities of patients, pulmonary spirometry and surgical approaches, which showed potential to be implemented universally (Brunelli et al., 2017).

The predictive models’ clinical benefits and public health significance encourage medical practitioners to evaluate their validity and reliability among surgery-treated lung cancer patients across different countries and ethnicities (Nagoya et al., 2019; Pompili et al., 2018; Taylor et al., 2020). To our knowledge, no studies have applied and validated these models in China. This study aims to externally validate five predictive models for postoperative cardiopulmonary morbidity in a Chinese population. Results of updated models would be presented as well. This article was presented in accordance with the TRIPOD reporting checklist.

## Materials & Methods

### Patient selection

A retrospective study was conducted of patients who underwent pulmonary resection at Peking Union Medical College Hospital from 2018/09/01 to 2019/08/31. Inclusion criteria included an age of at least 18 years old, anatomic pulmonary resection, systematic lymph node dissection or sampling, and no neoadjuvant therapy. Exclusion criteria included a postoperative pathological report suggesting a non-lung cancer mass and the absence of essential data, such as forced expiratory volume in 1 s (FEV1). It was a complete-case analysis. The study was conducted in accordance with the Declaration of Helsinki (as revised in 2013). This study was approved by the Institutional Review Board of Peking Union Medical College Hospital (No. S-K 1602). All patients provided written informed consent to participants in each medical record, and patient details were anonymized before analysis.

### Term definitions

Variables and outcomes were defined in previous studies (Brunelli et al., 2017; Fernandez et al., 2015; Nagoya et al., 2019). The postoperative cardiopulmonary complications included prolonged mechanical ventilation >24 h, pneumonia, atelectasis, airway stenosis, empyema, chylothorax, respiratory failure, pulmonary embolism, pulmonary edema, pneumothorax, prolonged air leak >5 days, bronchopleural fistula, reintubation, arrhythmia, acute myocardial infarction, acute kidney injury, stroke, postoperative bleeding, recurrent nerve palsy and phrenic nerve palsy (Brunelli et al., 2017; Nagoya et al., 2019). Apart from those, we noticed that increased pleural effusion that was nonchylous and nonpurulent occurred commonly in our center. It was not listed as a kind of postoperative cardiopulmonary complication in European and Japanese populations (Brunelli et al., 2020; Nagoya et al., 2019). Our center reported that increased nonchylous and nonpurulent effusion was one of the leading causes of delayed discharge (Liu et al., 2021). Therefore, a secondary analysis considering it as a kind of postoperative complication would also be performed.

### Models for validation

This study evaluated the performance on predicting postoperative cardiopulmonary morbidity of five existing models in a Chinese population, which were the logit form of Eurolung1 (2016E1) and parsimonious Eurolung1 (2019E1), the Brunelli model, the aggregate form of Eurolung1 (aE1) and the Age-adjusted Charlson Comorbidity Index (ACCI). Detailed information of the models is presented in the supplementary information. The first three were logistic models, while the left were aggregate models. The Brunelli model was developed by Brunelli et al. in 2006 and included age, the percentage of predicted forced vital capacity, extended resection and cardiac comorbidity as predictors (Brunelli et al., 2006). ACCI was a derivative of the Charlson Comorbidity Index, initially developed to predict 1-year mortality (Yang et al., 2018). Previous studies showed that ACCI had a better performance than the Charlson Comorbidity Index and Elixhauser comorbidity index in predicting survival in lung cancer patients (Yang et al., 2018). ACCI could also predict postoperative complications in pelvic surgeries, advanced primary epithelial ovarian cancer, and gastric cancer (Dessai et al., 2018; Kahl et al., 2017; Maezawa et al., 2019). Stamenovic et al. demonstrated that ACCI was a strong predictor for postoperative complications among patients over 70 years old, but seldom studies have investigated its efficacy among general lung cancer patients (Stamenovic, Messerschmidt & Schneider, 2018). Performance status, comorbidity score and New York Heart Association score were absent in our database, and thus we did not evaluate the performance of Thoracoscore and the modified Thoracoscore (Bradley et al., 2012).

### Statistical analysis

Numerical variables were presented as median and interquartile range, and categorical variables were presented as count and percentage. The predicted forced expiratory volume in 1 s and predicted forced vital capacity were calculated according to the equations established by Jian et al. (2017). Since they did not describe the parameters of the equations for patients over 81 years old, those parameters were estimated by linear regression. Model performance was assessed by discrimination, calibration and several characteristics, such as sensitivity (Farjah et al., 2015). Model discrimination was characterized by the area under the receiver operating characteristic curve (AUC). AUC>0.7 indicates good discrimination ability, while AUC>0.8 indicates strong discrimination ability. The calibration of logistic models was assessed by the Hosmer–Lemeshow test and calibration curves (Paul, Pennell & Lemeshow, 2012; Taylor et al., 2020). A non-significant *p* value of the Hosmer–Lemeshow test indicates a good fit. If the slope of a calibration curve is close to 1 and the intercept is close to 0, the model has a good fit. Specificity, sensitivity, negative predictive value (NPV), positive predictive value (PPV) and accuracy were calculated. Patients shall be dichotomized to describe them, but all models provided no thresholds. Consequently, the threshold was set at the Youden Index for both logistics and aggregate models, which was commonly observed (Youden, 1950). Three methods were applied to update the model. Method 1 was recalibration in the large, merely re-estimating the intercept. Method 2 was logistic recalibration, re-estimating the intercept and the slopes of the linear predictors. Method 3 re-estimated all regression coefficients, which was model revision (Vergouwe et al., 2017). Group differences were tested by the chi-square test or Fisher’s exact test. Differences of medians were tested by Wilcoxon signed-rank test (Li & Johnson, 2014). A *p* value <0.05 was considered statistically significant. All statistical analyses were carried out using the R Project for Statistical Computing version 4.0.2 (RRID:SCR_001905).

## Results

### Baseline demographics and clinical characteristics

**Figure 1:**
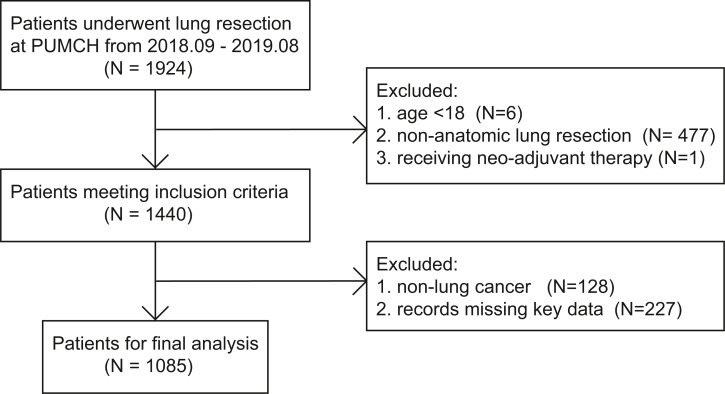
The flowchart of patient selection.

Among 1924 patients who underwent lung resection at our center from 2018/09/01 to 2019/08/31, 484 patients did not meet the inclusion criteria. A total of 128 patients had pathological reports suggesting non-lung cancer masses, 227 lacked critical data, and the remaining 1,085 patients were included in further analysis (Fig. 1).

Detailed clinical characteristics were shown in Table 1. Nearly all of the characteristics of Chinese patients were significantly different from those of European patients (Brunelli et al., 2020). We observed that the median age (60.0 *vs.* 64.6, *p* < 0.01), the percentage of males (38.99% *vs.* 65.28%, *p* < 0.01), the median BMI (23.9 *vs.* 25.1, *p* < 0.01), coronary artery disease (5.44% v.s 8.16%, *p* < 0.01), and chronic kidney disease (0.46% v.s 5.55%, *p* < 0.01) were significantly lower than those in the European population. The majority of patients received video-assisted thoracic surgery (VATS) (97.70% *vs* 25.65%, *p* < 0.01) and no extended resection (1.11% *vs* 5.73%, *p* < 0.01). The numbers of patients receiving segmentectomy, lobectomy, bilobectomy and pneumonectomy were 210, 863, 11 and 1, respectively (Brunelli et al., 2020). The median of the percentage of predicted postoperative forced expiratory volume in 1 s (ppoFEV1%) of our patients was significantly higher than that of European patients (76% *vs.* 73%, *p* < 0.01).

**Table 1:** Patient characteristics of the Chinese population and the European population.

Characteristics	Chinese population	European population	*p*
Age			<0.01
median (IQR)	60.0 (52.0–65.0)	64.6 (57.6–71.2)	
Sex (%)			<0.01
Male	423 (38.99)	53780 (65.28)	
Female	662 (61.01)	28603 (34.72)	
Body mass index (kg/m^2^)			<0.01
median (IQR)	23.9 (22.0–26.1)	25.1 (22.4–28.3)	
Postoperative FEV1%			<0.01
median (IQR)	76 (68–86)	73 (59–87)	
FVC%			–
median (IQR)	88.91 (79.87–98.52)	–	
Medical history (%)			
Coronary artery disease	59 (5.44)	6725 (8.16)	<0.01
Cerebrovascular disease	23 (2.12)	2434 (2.95)	0.13
Chronic kidney disease	5 (0.46)	4579 (5.55)	<0.01
Arrhythmia	23 (2.12)	–	–
Chronic obstructive pulmonary disease	41 (3.78)	–	–
Diabetes mellitus	130 (11.98)	–	–
Hypertension	312 (28.76)	–	–
Smoking	235 (21.66)	–	–
Alcohol consumption	139 (12.81)	–	–
Approach (%)			<0.01
VATS	1060 (97.70)	21131 (25.65)	
Thoracotomy	25 (2.30)	61252 (74.35)	
Type of resection (%)			<0.01
Segmentectomy	210 (19.35)	7418 (9.00)	
Lobectomy	863 (79.54)	63681 (77.3)	
Bilobectomy	11 (1.01)	3617 (4.49)	
Pneumonectomy	1 (0.00)	7667 (9.31)	
Extended resection (%)			<0.01
Yes	1073 (98.89)	77611 (94.27)	
No	12 (1.11)	4772 (5.73)	

**Notes.**

IQR interquartile range FEV1% the percentage of predicted forced expiratory volume in 1 s FVC% the percentage of predicted forced vital capacity VATS video-assisted thoracic surgery

Ninety-one patients experienced postoperative cardiopulmonary morbidities defined by the European Society of Thoracic Surgeons (ESTS). Among them, 1 patient had 3 kinds of complications. Another ninety-four patients experienced increased pleural effusion, which cannot be categorized into empyema or chylothorax (Table 2).

**Table 2:** Details of postoperative cardiopulmonary morbidity.

Types	Numbers of events (%)
Terms mentioned by Brunelli et al. and Nagoya et al.	
Prolonged mechanical ventilation	2 (0.18)
Pneumonia	5 (0.46)
Atelectasis	4 (0.37)
Empyema	2 (0.18)
Chylothorax	7 (0.65)
Respiratory failure	2 (0.18)
Pulmonary embolism	1 (0.09)
Arrhythmia	2 (0.18)
Acute myocardial infarction	3 (0.28)
Acute kidney injury	1 (0.09)
Prolonged air leak	61 (5.62)
Postoperative bleeding	1 (0.09)
Others	
Increased pleural effusion	94 (8.66)
Total cases	185

### Model performance

**Figure 2:**
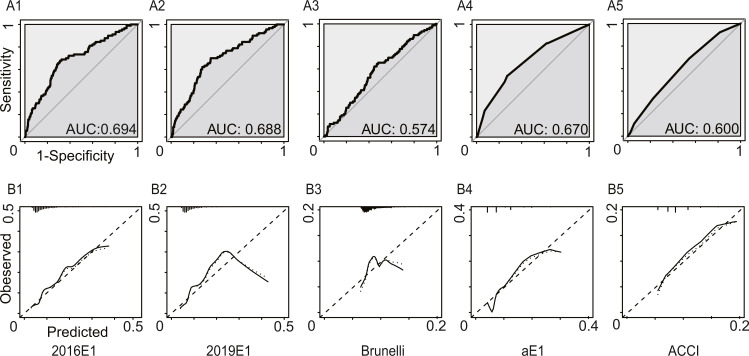
The model performances in the first analysis. Postoperative cardiopulmonary complications were defined by the European Society of Thoracic Surgeons. (A) The receiver operating characteristic curves (ROC) and the area under the ROC curve (AUC), indicating discrimination ability. (B) Calibration plots. 2016E1, the logit form of Eurolung1; 2019E1, the logit form of parsimonious Eurolung1; Brunelli, the Brunelli model; aE1, the aggregate form of Eurolung1; ACCI, the Age-adjusted Charlson Comorbidity Index.

Figure 2 and Table 3 summarized the model performance. In this analysis, only morbidities defined by ESTS were calculated. The AUC values ranged from 0.574 (95% CI [0.515–0.633]) for the Brunelli model to 0.694 (95% CI [0.636–0.753]) for the 2016E1 model. 2019E1 and aE1 also achieved AUC values similar to that of 2016E1, which were slightly lower than 0.70. This result indicated acceptable but not good enough discrimination ability. For calibration of logistic models, only 2019E1 achieved a good fit, while others were miscalibrated. The calibration of aE1 and ACCI was not tested. The calibration curve of 2019E1 indicated that it over-predicted the risk of morbidities in the relatively high-risk group. 2019E1 had the highest sensitivity (0.700), while aE1 had the highest specificity (0.728). The PPV values of all models were low (range: 0.103−0.164), but the NPV values were high (range: 0.942−0.960).

**Table 3:** Model performances in the first analysis.

	2016E1	2019E1	Bruneli	aE1	ACCI
AUC	0.694	0.688	0.574	0.670	0.600
95% CI	0.636–0.753	0.630–0.745	0.515–0.633	0.613–0.728	0.543–0.657
Goodness-of-fit test (*P* value)	<0.01	0.23	<0.01	–	–
Performance characteristics					
Sensitivity	0.678	0.700	0.644	0.544	0.689
Specificity	0.688	0.649	0.531	0.728	0.456
PPV	0.164	0.153	0.110	0.153	0.103
NPV	0.959	0.960	0.943	0.946	0.942
Accuracy	0.688	0.653	0.540	0.712	0.476

**Notes.**

2016E1 the logit form of Eurolung1 2019E1 the logit form of parsimonious Eurolung1 Bruneli the Bruneli model aE1 the aggregate form of Eurolung1 ACCI the Age-adjusted Charlson Comorbidity Index AUC area under the receiver operating characteristic curve CI confidence interval PPV positive predictive value NPV negative predictive value

### Model performance in the secondary analysis

**Figure 3:**
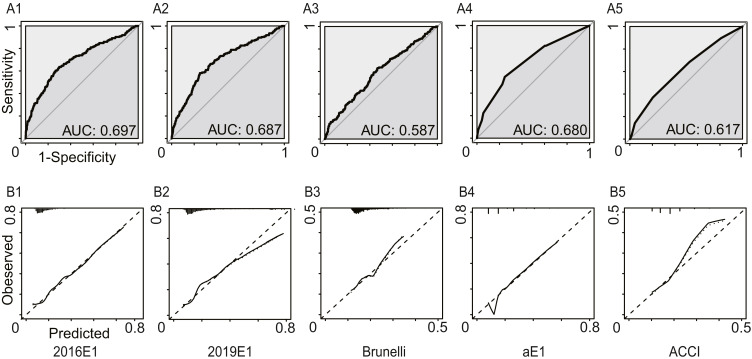
The model performances in the secondary analysis. Increased pleural effusion was further regarded as a kind of postoperative cardiopulmonary complication. (A) The receiver operating characteristic curves (ROC) and the area under the ROC curve (AUC), indicating discrimination ability. (B) Calibration plots. 2016E1, the logit form of Eurolung1; 2019E1, the logit form of parsimonious Eurolung1; Brunelli, the Brunelli model; aE1, the aggregate form of Eurolung1; ACCI, the Age-adjusted Charlson Comorbidity Index.

In this analysis, we regarded increased pleural effusion as a kind of postoperative cardiopulmonary complication. Figure 3 and Table 4 summarized the model performance. Similarly, the AUC values ranged from 0.587 (95% CI [0.541–0.633]) for the Brunelli model to 0.697 (95% CI [0.652–0.742]) for the 2016E1 model, which showed non-significant improvement compared to those in the first analysis. However, all logistic models had *p* values of goodness-of-fit test <0.01, suggesting poor calibration ability. 2016E1 had the highest sensitivity (0.636), while ACCI had the highest specificity (0.796). The PPV values of all models were relatively low (range: 0.216−0.321), but the NPV values were relatively high (range: 0.861−0.904).

**Table 4:** Model performances in the secondary analysis.

	2016E1	2019E1	Bruneli	aE1	ACCI
AUC	0.697	0.687	0.587	0.680	0.617
95% CI	0.652–0.742	0.644–0.731	0.541–0.633	0.638–0.723	0.573–0.661
Goodness-of-fit test (*P* value)	<0.01	<0.01	<0.01	–	–
Performance characteristics					
Sensitivity	0.636	0.582	0.630	0.538	0.370
Specificity	0.701	0.749	0.532	0.755	0.796
PPV	0.303	0.321	0.216	0.309	0.270
NPV	0.904	0.898	0.876	0.889	0.861
Accuracy	0.690	0.721	0.548	0.718	0.724

**Notes.**

2016E1 the logit form of Eurolung1 2019E1 the logit form of parsimonious Eurolung1 Bruneli the Bruneli model aE1 the aggregate form of Eurolung1 ACCI the Age-adjusted Charlson Comorbidity Index AUC area under the receiver operating characteristic curve CI confidence interval PPV positive predictive value NPV negative predictive value

### Model updating

A prediction model applying to a new clinical setting may present calibration drifting. Our study indicated that nearly all models had relatively poor calibration performance. Three logistic regression models were under consideration for model updating. The Brunelli model had the poorest discrimination ability, making model updating less meaningful. The 2016E1 model which had the highest AUC value but poor calibration ability among them was selected for model updating. As shown in Table 5, all updated models exerted adequate calibration performance (*p* > 0.05). The AUC values slight increased and achieved 0.703 in the revised model updated by method 3. Although the 2019E1 model achieved a good fit, we still updated it by three methods and obtained similar outcomes as the original 2019E1 did (Table S1). Calibration plots were showed in Figs. S1 and S2.

**Table 5:** Model updating of the 2016E1 model.

	2016E1			
	Original	Method 1	Method 2	Method 3
Coefficients				
Age	0.026	0.026	0.035	0.017
Sex	0.497	0.497	0.666	0.906
ppoFEV1%	−0.015	−0.015	−0.020	−0.028
Thoracotomy	0.497	0.497	0.666	−0.917
Extended resection	0.514	0.514	0.689	0.898
CAD	0.231	0.231	0.309	0.365
CVD	0.371	0.371	0.497	1.449
CKD	0.152	0.152	0.204	1.543
Intercept	−2.465	−3.119	−3.412	−1.914
AUC	0.694	0.695	0.695	0.703
95% CI	0.636–0.753	0.636–0.753	0.636–0.753	0.644–0.762
Goodness-of-fit test (*P* value)	<0.01	0.076	0.159	0.493

**Notes.**

2016E1 the logit form of Eurolung1 ppoFEV1% the percentage of predicted postoperative forced expiratory volume in 1 s CAD coronary artery disease CVD cerebrovascular disease CKD chronic kidney disease AUC area under the receiver operating characteristic curve CI confidence interval

## Discussion

In the present study, we evaluated the performance of five models predicting postoperative cardiopulmonary morbidities after thoracic surgery. For original models, the logit form of the parsimonious Eurolung1 model (2019E1) showed acceptable discrimination ability (AUC 0.688, 95% CI [0.630–0.745]) and good calibration ability simultaneously, while other had poor discrimination or calibration ability. After model updating, the logit form of the Eurolung1 model (2016E1) also had acceptable discrimination ability (AUC 0.695−0.703) and a good fit (*p* > 0.05).

Several methods were applied to improve the accuracy of validation. First, we applied new equations derived originally from Chinese patients to calculate the predicted forced expiratory volume in 1 s and predicted forced vital capacity based on the results of Jian et al. They showed that the new equations had better performance in providing predicted values than the SEA-GLI2012, NEA-GLI2012, Asian-NHANESIII and Chinese-ECSC 1993 equations, which were widely used in mainland China (Jian et al., 2017). It facilitated precise calculations of probabilities. Second, we carefully chose the number of groups (g) of the Hosmer–Lemeshow test. Typically, g was set as 10. Paul, Pennell & Lemeshow (2012) found that the power of the Hosmer–Lemeshow test decreased with g and offered the following formula to select an appropriate g for cohorts with 1000 <cases ≤ 25000: $g=\max [10,\min \left( \frac{m}{2} , \frac{n-m}{2} ,2+8\ast \frac{{n}^{2}}{1000000} \right) ]$, where n = the number of cases and m = the number of events. Accordingly, we used *g* = 11 for the Hosmer–Lemeshow test. Third, all patients had complete data.

External validation of models in a population with different backgrounds is clearly warranted to evaluate model generalizability, but the discrepancy always makes it challenging. Nagoya et al. (2019) demonstrated that Eurolung models for morbidity and mortality prediction could not be applied to a Japanese population due to the discrepancy of several baseline characteristics between the Japanese population and the European population. Taylor et al. (2020) validated 6 models for short-term mortality prediction in UK patients, which included Eurolung models, the Brunelli model, Thoracoscore and the European Society Objective Score. They found that parsimonious Eurolung2 had good discrimination and calibration (AUC 0.73, p for O:E ratio >0.05), while other models showed inadequate performance. Apart from models of thoracic surgery, a similar situation existed in other fields. In cardiac surgery, EuroScore II showed good performance in Chinese patients receiving coronary artery bypass grafting but underpredicted mortality rates in the high-risk subgroup (Bai et al., 2016). EuroScore II was suitable for predicting the operative risk in Chinese patients undergoing single valve surgery, but it could not achieve the same performance in patients receiving multiple valve surgery (Wang et al., 2016). Moreover, both the STS score and the Parsonnet model had poor calibration when predicting prolonged intense care unit stay (*p* value for the Hosmer–Lemeshow test <0.001) among Chinese patients receiving heart valve surgery (Zhang et al., 2011).

Overall, these models did not fit well among this Chinese population. The logit form of the parsimonious Eurolung1 (2019E1) was the best-performing model in predicting postoperative cardiopulmonary morbidity. However, its AUC value only achieved an acceptable level (0.688, 95% CI [0.630–0.745]). There might be two main reasons. The first is the discrepancy in patient characteristics. As shown in Table 1, age, sex, comorbidities (*e.g.* chronic kidney disease), surgical approaches and extent of resection differed between the two populations. Age was listed as an important predictor in all models tested. Many studies have shown that advanced age and male sex were associated with postoperative cardiopulmonary complications (Nakada et al., 2019; Sezen et al., 2019; Zhang et al., 2017). Preoperative comorbidities affect the general health of patients. Benker et al. suggested that coronary artery disease and hypertension were associated with cardiac complications, while smoking was related to pulmonary complications (Benker et al., 2021). More patients in our center had early-stage lung cancer, demonstrated by a larger percentage of segmentectomy and VATS. Accumulated studies have revealed that VATS results in lower postoperative morbidity, shorter length of hospital stay, less postoperative pain and better quality of life than thoracotomy (Bendixen et al., 2016; Bendixen et al., 2019). In addition, the Eurolung1 model did not fit well among Japanese patients, mainly explained by the discrepancy in patient characteristics (Nagoya et al., 2019). The second reason may be the inherent characteristics of the models. 2016E1 and 2019E1 achieved AUC values of 0.711 and 0.710 in 82383 European patients, which were regarded as common and acceptable outcomes of risk models in the cardiothoracic area (Brunelli et al., 2020). Brunelli et al. explained it by the thought that uncaptured variables might have potential associations with outcomes (Brunelli et al., 2017). The Brunelli model was developed based on a relatively small sample size (*n* = 1062) in 2006, which may lead to selection bias (Brunelli et al., 2006). In addition, the rapid development of medical science in the past decade has affected model performance to some extent. ACCI could reflect the preoperative physical status, and thus it has the potential to predict postoperative morbidity. However, it failed to perform well. An explanation was that ACCI was not specific enough due to the paucity of lung cancer-related predictors, such as the surgical approach and ppoFEV1%. They were important indicators of postoperative complications (Bendixen et al., 2016; Zhang et al., 2015).

In the secondary analysis, increased pleural effusion was labeled a kind of postoperative cardiopulmonary complication. Although the AUC values slightly increased, all logistic models were miscalibrated. Therefore, we do not recommend this change.

Calibration drift is a common phenomenon when a logistic regression model is applied in a different population. Conventionally, model recalibration, model revision and model extension can be used. Model recalibration re-estimates the intercept and/or the slope of the linear predictor. Model revision re-estimates all coefficients, while model extension adds new predictors. Some studies suggested that simple updating methods should be preferred, such as model recalibration (Steyerberg et al., 2004; Vergouwe et al., 2017). More extensive model revisions can be considered if the validation dataset is relatively large. Updated by model recalibration and revision, the new three models of 2016E1 showed adequate calibration and small improvements in discrimination, which was similar with previous studies focused on validation and recalibration (Harrison et al., 2015; Wessler et al., 2017). Nevertheless, the discrimination ability was merely acceptable, leaving the possibility to develop new models de novo.

As stated above, developing risk models using large databases may neglect some important variables that were not calculated or captured, which may impair model performance. For their initial analysis, most models evaluated in this study did not include a history of smoking, alcohol consumption, hypertension, chronic obstructive pulmonary disease or arrhythmia. However, they occurred in a relatively large proportion of our patients (shown in Table 1), which may lead to differences. These preoperative variables should be considered. Another way to improve model performance is to apply machine learning analytics instead of logistic regression during model development. Machine learning analytics may find complex relationships in data and accommodate nonlinear issues, which could provide more accurate models (Zhang, Ho & Hong, 2019; Zhang et al., 2020).

This study had several limitations. It was a single-center retrospective study. This may have unavoidable selection bias, and the conclusions could not be directly applied to the whole Chinese population. For example, the majority of patients in our study had early-stage lung cancer and received VATS. Our results may not be extrapolated to the population consisting of more advanced lung cancer patients and centers that carry out thoracotomy more often. However, the popularization of routine health checks and VATS allows an increasing number of people to detect early-stage disease and minimize the negative influence of surgeries, which makes our results meaningful. The calibration ability of aE1 and ACCI were not tested by the Hosmer–Lemeshow test. One reason was that this test was developed for logistic models. The aE1 model was directly derived from 2016E1, and thus aE1’s performance would be comparable to 2016E1. ACCI achieved an AUC value of 0.600, indicating relatively low discrimination ability, which made the calibration test less meaningful.

An accurate prediction model is of significance in clinical practice. Our long-term goal is to provide evidence-based suggestions for Chinese thoracic surgeons to better weigh the risk of surgery and tumor progression, and integrate the considerations of quality of life for operable patients. A good model could assist thoracic surgeons in more accurately personalizing operation schemes to achieve patient-centered care and navigate during the preoperative shared decision-making process. In addition, establishing a universal model could further benefit the administration and improvement of clinical practice across hospitals in the whole country.

## Conclusions

Overall, these models did not show good performance in our population. The original parsimonious Eurolung1 and the updated Eurolung1 were the best-performing models on morbidity prediction, but their discrimination ability only achieved an acceptable level. A multicenter study is warranted to develop new models among Chinese populations in the future. More preoperative variables and sophisticated statistical methods should be taken into consideration when developing models.

##  Supplemental Information

10.7717/peerj.12936/supp-1 Supplemental Information 1 The detailed information of predictive models validated in this study

10.7717/peerj.12936/supp-2 Supplemental Information 2 Information on 1085 patients analyzed in this study

10.7717/peerj.12936/supp-3 Supplemental Information 3 Model updating of the 2019E1 model

10.7717/peerj.12936/supp-4 Supplemental Information 4 Calibration plots of the original and updated 2016E1 models These plots show the calibration of (A) the original model, (B) the model updated by method 1 of re-estimating the intercept, (C) the model updated by method 2 of re-estimating the intercept and the slope of linear predictor, and (D) the model updated by method 3 of re-estimating all coefficients. 2016E1, the logit form of Eurolung 1.

10.7717/peerj.12936/supp-5 Supplemental Information 5 Calibration plots of the original and updated 2019E1 models These plots show the calibration of (A) the original model, (B) the model updated by method 1 of re-estimating the intercept, (C) the model updated by method 2 of re-estimating the intercept and the slope of the linear predictor, and (D) the model updated by method 3 of re-estimating all coefficients. 2019E1, the logit form of parsimonious Eurolung 1.

## Additional Information and Declarations

### Data Availability

The following information was supplied regarding data availability: The raw data is available in the Supplemental File.
