# Supplementary material for: External validation of five predictive models for postoperative cardiopulmonary morbidity in a Chinese population receiving lung resection
Source: PeerJ. 2022 Feb 9;10:e12936. doi: 10.7717/peerj.12936 (PMC8840067; doi:10.7717/peerj.12936)
Supplement: Supplemental Information 1 [file peerj-10-12936-s001.pdf]

Supplementary Appendix

The Eurolung1 model(Brunelli et al. 2017):
logit (morbidity) = -2.465 + 0.497*sex male (coded 1 for male and 0 for female) + 0.026*age + 0.231*CAD (coded 1 for presence of CAD) + 0.371*CVD (coded 1 for presence of CVD) + 0.152*CKD (coded 1 for presence of CKD) - 0.015*ppoFEV1 + 0.514*extended resections[#] (coded 1 for presence of extended resection) + 0.497*thoracotomy (coded 1 for thoracotomy and 0 for VATS)

The parsimonious Eurolung1 model(Brunelli et al. 2020):
logit (morbidity) = -2.852 + 0.021*age + 0.472*male - 0.015*ppoFEV1 + 0.662*thoracotomy + 0.324* extended resection[#]

The aggregate Eurolung1 model(Brunelli et al. 2017):
1 point: CKD
2 points: CAD and CVD
3 points: age > 65, sex male, thoracotomy, extended resections[#] and ppoFEV1 < 70%
The points were summed for each patient to generate an aggregate score.

[#]extended resection: associated with chest wall, Pancoast tumours, resection of the atrium or superior vena cava, diaphragm resection, vertebral resection, pleuropneumonectomy, sleeve pneumonectomies, intrapericardial pneumonectomy

The Brunelli model(Brunelli et al. 2006):
Predicted morbidity: ln [R/(1-R)] = -2.1 + 0.035*age - 0.02*FVC + 0.6*extended resection + 0.7*cardiac co-morbidity
FVC: the percentages of predicted forced vital capacity
cardiac co-morbidity: previous cardiac surgery, previous myocardial infarction, history of coronary artery disease, current treatment for arrhythmia, cardiac failure or hypertension.

The Age-adjusted Charlson comorbidity index(Yang et al. 2018):
1 point: Myocardial infarction, congestive heart failure, peripheral vascular disease, cerebral vascular disease, dementia, chronic obstructive pulmonary disease, connective tissue disease, ulcer disease, mild liver disease, diabetes
2 points: Hemiplegia, moderate/severe renal disease, diabetes with end-organ damage, any tumor, leukemia, lymphoma
3 points: Moderate/severe liver disease
6 points: Metastatic solid tumor, AIDS
Additional: 1 point for each decade over age 40 years

Reference

Brunelli A, Cicconi S, Decaluwe H, Szanto Z, and Falcoz PE. 2020. Parsimonious Eurolung risk models to predict cardiopulmonary morbidity and mortality following anatomic lung resections: an updated analysis from the European Society of Thoracic Surgeons database. *Eur J Cardiothorac Surg* 57:455–461. 10.1093/ejcts/ezz272

Brunelli A, Salati M, Rocco G, Varela G, Van Raemdonck D, Decaluwe H, and Falcoz PE. 2017. European risk models for morbidity (EuroLung1) and mortality (EuroLung2) to predict outcome following anatomic lung resections: an analysis from the European Society of Thoracic Surgeons database. *Eur J Cardiothorac Surg* 51:490–497. 10.1093/ejcts/ezw319

Brunelli A, Xiume F, Al Refai M, Salati M, Marasco R, and Sabbatini A. 2006. Risk-adjusted morbidity, mortality and failure-to-rescue models for internal provider profiling after major lung resection. *Interact Cardiovasc Thorac Surg* 5:92–96. 10.1510/icvts.2005.118703

Yang CC, Fong Y, Lin LC, Que J, Ting WC, Chang CL, Wu HM, Ho CH, Wang JJ, and Huang CI. 2018. The age-adjusted Charlson comorbidity index is a better predictor of survival in operated lung cancer patients than the Charlson and Elixhauser comorbidity indices. *Eur J Cardiothorac Surg* 53:235–240. 10.1093/ejcts/ezx215
