# Supplementary material for: External validation of five predictive models for postoperative cardiopulmonary morbidity in a Chinese population receiving lung resection
Source: PeerJ. 2022 Feb 9;10:e12936. doi: 10.7717/peerj.12936 (PMC8840067; doi:10.7717/peerj.12936)
Supplement: Supplemental Information 3 [file peerj-10-12936-s003.docx]

**Table S1** Model updating of the 2019E1 model

|  | 2019E1 | | | |
| --- | --- | --- | --- | --- |
|  | Original | Method 1 | Method 2 | Method 3 |
| Coefficients |  |  |  |  |
| Age | 0.021 | 0.021 | 0.028 | 0.023 |
| Sex | 0.472 | 0.472 | 0.639 | 0.946 |
| ppoFEV1% | -0.015 | -0.015 | -0.020 | -0.029 |
| Thoracotomy | 0.662 | 0.662 | 0.896 | -0.961 |
| Extended resection | 0.324 | 0.324 | 0.438 | 0.785 |
| Intercept | -2.852 | -2.776 | -2.949 | -2.105 |
| AUC | 0.688 | 0.688 | 0.688 | 0.694 |
| 95% CI | 0.630-0.745 | 0.630-0.745 | 0.630-0.745 | 0.637-0.752 |
| Goodness-of-fit test (P value) | 0.23 | 0.31 | 0.54 | 0.24 |

2019E1, the logit form of parsimonious Eurolung1; ppoFEV1%, the percentage of predicted postoperative forced expiratory volume in 1 second; AUC, area under the receiver operating characteristic curve; CI, confidence interval
